# Volatile organic compounds in exhaled breath of newborns: a pilot study

**DOI:** 10.1038/s41372-024-02102-2

**Published:** 2024-08-28

**Authors:** Mohsen A. A. Farghaly, Somaya Abuelazm, Marwa M. Elgendy, David Grove, Jalal M. Abu-shaweesh, Raed A. Dweik, Hany Aly

**Affiliations:** 1 Neonatology Division, Cleveland Clinic Children’s Hospital, Cleveland, OH USA; 2 Respiratory Institute, Cleveland Clinic, Cleveland, OH USA; 3 Lerner Research Institute, Cleveland Clinic, Cleveland, OH USA

**Keywords:** Predictive markers, Diagnostic markers, Metabolism

## Abstract

**Objective:**

To assess volatile organic compounds (VOCs) in breath samples collected non-invasively from preterm and full-term infants.

**Methods:**

This was a pilot study included preterm and full-term infants who were not intubated or suspected or diagnosed with metabolic or gastrointestinal disorders. The samples were analyzed for VOCs using a selected-ion flow-tube mass spectrometer.

**Results:**

Twenty infants were included; ten preterm and ten full-term infants. Twenty-two VOCs were detected and measurable in all samples. There was a significant difference between preterm and full-term infants for the 2-propanol, acetaldehyde, acetone, acetonitrile, benzene, ethanol, isoprene, pentane, 3-methylhexane, 2-nonene, ethane, triethylamine, and trimethylamine compounds.

**Conclusion:**

It is feasible to measure VOCs in breath samples of preterm and full-term non-intubated infants. Full-term infants express different concentrations than preterm infants. Further studies are needed to examine the utility and reproducibility of measuring VOCs to identify neonatal diseases and predict outcomes.

## Introduction

Metabolites are low-molecular-weight compounds that represent the ultimate end products of gene and protein expression. The global collection of metabolites, known as the metabolome, that maintains dynamic homeostasis and helps determine minute-to-minute cellular phenotype [1]. Recent advances in metabolite analysis allow for simultaneous measurement of many metabolites from biologic samples. This broad metabolic analysis can help define unique metabolic patterns and affected pathways present in specific patient groups or disease states [2]. Numerous metabolic processes in the body produce end products which are dissolved in the blood and are excreted by the lungs. Exhaled breath constitutes a complex mixture of hundreds of metabolites, volatile organic compounds (VOCs), that could potentially be used as a safe and noninvasive method of diagnostic and therapeutic monitoring. In adults, human studies showed associations of VOCs with shock, stroke, brain injury, heart failure and liver cirrhosis using breath test for metabolomics screening [3–9].

In infants, many studies were able to measure VOCs in stool or urine samples [10–15]. It is not known if VOCs can be collected accurately and be reflective of body metabolism from breath samples of non-intubated infants, and whether there is a difference between preterm and full-term infants. We hypothesized that VOCs, if feasible to measure, would differ among preterm and full-term infants. If proven feasible, we envision using this technology to predict, and ultimately ameliorate complications of prematurity and other neonatal conditions. Thus, we aimed in this feasibility study to assess VOCs in breath samples collected non-invasively from preterm and full-term infants.

## Methods

This pilot study was conducted at the Neonatal Intensive Care Unit (NICU) at the Cleveland Clinic Children’s Hospital. The study was approved by the Cleveland Clinic’s Institute Review Board (IRB). Parental consents were obtained before any subject recruitment.

The study included newborn infants admitted to the NICU. Infants were included if they were born at preterm age (<30 weeks of gestational age (GA)). Infants were included only if they were not intubated nor receiving invasive mechanical ventilation and successfully achieved full enteral feeds for at least 24 h. Infants were excluded if they were intubated and/or supported with invasive mechanical ventilation, receiving parenteral nutrition or not on full feeds, and/or infants with suspected or diagnosed infection, metabolic or gastrointestinal disorders. A reference group of full-term infants (≥37 weeks of GA) were also included. Clinical and demographic data of enrolled infants included GA, postnatal age, race, birth weight, current weight, enteral feeding {formula or breast milk}, respiratory support {none, nasal canula (NC), continuous positive airway pressure (CPAP), high flow nasal canula (HFNC), noninvasive nasal intermittent ventilation (NIMV), or non-invasive-neurally-adjusted ventilatory assist (NIV-NAVA)}, and how much FiO_2_.

A fifty milliliters of exhaled breath condensate sample were collected from the expiratory side of the breathing tubes for each infant using dissolved gas analysis glass syringe (capacity 50 mL, graduated, 2 mL, tip style, needle-lock Luer, model CADG5157-1EAMKCS3025) (Sigma-Aldrich of Merck KGaA, Darmstadt, Germany). During sample collection investigators did not have a physical contact with the infant. The investigator put on full protective equipment including disposable gowns, gloves, goggles, and masks. All patients were on cardiorespiratory monitors and registered nurse and respiratory therapist bedside during sample collection. After obtaining the sample, the syringe was capped and was put in a non-collapsible box for safe transport for immediate processing and analysis. If the full-term control infant did not require respiratory support, sample was directly obtained from the ambient air inside the incubator.

The samples were analyzed for VOCs using a selected-ion flow-tube mass spectrometer (SIFT-MS), the model used was Voice 200 ultra from SYFT Technologies. The SIFT-MS instrument was run in the selected-ion mode (SIM); this is where the concentration of the VOCs is measured by monitoring the product ion peaks. The compounds monitored were 2-propanol, acetaldehyde, acetone, acetonitrile, acrylonitrile, benzene, carbon disulfide, dimethyl sulfide, ethanol, isoprene, pentane, 1-decene, 1-heptene, 1-nonene, 1-octene, 3-methylhexane, 2-nonene, ammonia, ethane, hydrogen sulfide, triethylamine and trimethylamine. They were selected for their common presence in exhaled breath. All samples were analyzed by the same investigator (DG) who was blinded to the infant’s group or clinical condition.

Statistical analysis was conducted using the Mann-Whitney U (Wilcoxon) test to compare continuous variables. The Independent Sample t-test and Monte Carlo exact test were used to compare the clinical characteristics between the two groups. All analyses were performed using commercially available software (JMP pro version 14.0.0, SAS Institute, Inc, Cary, North Carolina; IBM SPSS Statistics version 21, IBM Corp, Armonk, New York). The level of significance was set at *P* < 0.05.

## Results

The study included twenty infants; ten preterm infants and ten full-term infants. Clinical and demographic data of enrolled infants are presented in Table 1. All preterm infants had respiratory distress and were receiving non-invasive respiratory support at time of enrollment. For the full-term infants’ group, seven infants did not require respiratory support. Preterm infants required higher FiO_2_ (*P* = 0.032). Average GA for preterm infants’ group was 25 weeks, while the average GA for the full-term infants’ group was 38 weeks, Table 1. There was no difference between the two groups based on race, 50% of preterm infants and 40% of full-term infants were White. Regarding type of enteral feeding, six preterm infants and 3 full-term infants were receiving breast milk, and the rest were receiving milk formula (*P* = 0.17).

**Table 1:** Characteristics of the study population (*n* = 20).

	GA	Postnatal age	Race	Birth weight	Current weight	Feeding	Respiratory support	FiO_2_
Patient 1	24 + 2	37	White	541	1388	Formula	CPAP	21
Patient 2	23 + 1	29	Black	500	970	Formula	CPAP	21
Patient 3	28 + 2	13	Other	1255	1278	Breast milk	CPAP	21
Patient 4	28 + 1	14	Other	1190	1260	Breast milk	CPAP	23
Patient 5	26 + 4	20	Black	1162	1180	Breast milk	NIV-NAVA	30
Patient 6	27 + 2	9	White	1152	1030	Breast milk	CPAP	35
Patient 7	28 + 2	22	White	1255	1458	Breast milk	HFNC	21
Patient 8	26 + 3	10	Black	1160	1121	Breast milk	CPAP	21
Patient 9	23 + 1	54	White	550	1700	Formula	NIMV	30
Patient 10	23 + 1	51	White	581	1380	Formula	HFNC	35
Patient 11	41 + 1	6	White	3130	3194	Breast milk	NC	21
Patient 12	39 + 5	7	Black	3180	3200	Breast milk	None	21
Patient 13	40 + 2	6	White	3090	3070	Breast milk	None	21
Patient 14	37 + 1	28	White	2820	3213	Formula	None	21
Patient 15	38 + 0	20	Black	2900	3150	Formula	None	21
Patient 16	37 + 3	22	White	2870	3210	Formula	None	21
Patient 17	39 + 0	7	Hispanic	2650	2700	Formula	NC	21
Patient 18	38 + 5	8	Hispanic	2720	2810	Formula	HFNC	24
Patient 19	39 + 1	6	Asian	2690	2730	Formula	None	21
Patient 20	39 + 0	8	Hispanic	2660	2730	Formula	None	21

*GA* gestational age in weeks + days, Postnatal age in days, Weight in grams. *FiO*_*2*_ fraction of inspired oxygen, in percentage.

*CPAP* continuous positive airway pressure, *NC* nasal cannula, *HFNC* high flow nasal canula, *NIMV* noninvasive nasal intermittent ventilation, *NIV-NAVA* non-invasive-neurally-adjusted ventilatory assist.

VOCs were detected in all collected samples (n = 20). Twenty-two VOCs were measurable in all subjects, namely, 2-Propanol, Acetaldehyde, Acetone, Acetonitrile, Acrylonitrile, Benzene, Carbon disulfide, Dimethyl sulfide, Ethanol, Isoprene, Pentane, 1-Decene, 1-Heptene, 1-Nonene, 1-Octene, 3-Methylhexane, 2-Nonene, Ammonia, Ethane, Hydrogen sulfide, Triethylamine, Trimethylamine, Table 2. Overall, there was a significant difference between preterm and full-term infants for the 2-propanol, acetaldehyde, acetone, acetonitrile, benzene, ethanol, isoprene, pentane, 3-methylhexane, 2-nonene, ethane, triethylamine and trimethylamine compounds, Table 2. The highest concentration of VOCs in both groups were observed in 2-Propanol, Acetaldehyde and 1-Heptene. When compared to full-term, preterm infants had higher concentration of one compound, 3-Methylhexane (*P* = 0.04). Full-term infants had higher concentrations of twelve compounds; 2-propanol, acetaldehyde, acetone, acetonitrile, benzene, ethanol, isoprene, pentane, 2-nonene, ethane, triethylamine and trimethylamine. The rest of the VOCs (nine compounds) did not differ in concentration between the two groups.

**Table 2:** Differences in VOCs concentration (PPM) between preterm and full-term infants.

	Preterm (*n* = 10)	Full-term (*n* = 10)	*P*-value*
• 2-Propanol	202 (174)	778 (453)	=0.017
• Acetaldehyde	24 (30)	59 (66)	=0.009
• Acetone	21 (32)	94 (104)	=0.007
• Acetonitrile	0.3 (0.1)	0.7 (0.4)	=0.035
• Acrylonitrile	0 (0.1)	0.1 (0.2)	=0.247
• Benzene	4.7 (2)	7.9 (3)	=0.009
• Carbon disulfide	4.6 (6)	4.7 (4)	=0.529
• Dimethyl sulfide	0.4 (0.3)	0.8 (0.9)	=0.123
• Ethanol	229 (245)	2921 (509)	<0.001
• Isoprene	0.7 (0.6)	1.6 (2)	=0.003
• Pentane	15 (7)	34 (22)	<0.001
• 1-Decene	5.3 (2.5)	4.3 (2.8)	=0.912
• 1-Heptene	95 (28)	84 (39)	=0.648
• 1-Nonene	37 (54)	39 (10)	=0.853
• 1-Octene	17 (9)	17.5 (5)	=0.989
• 3-Methylhexane	27 (28)	18 (8)	=0.043
• 2-Nonene	4.2 (4)	6.8 (3)	=0.039
• Ammonia	19 (13)	23 (8)	=0.097
• Ethane	1.9 (4)	3.2 (3)	=0.047
• Hydrogen sulfide	0 (0.3)	0 (0)	=0.681
• Triethylamine	1.06 (0.7)	1.09 (0.6)	=0.853
• Trimethylamine	7.5 (3.5)	15.5 (14)	=0.007

Data are expressed in median (IQR).

*PPM* parts per million.

^*^Mann Whitney U-test was used to compare the difference in median between groups.

## Discussion

This pilot study demonstrated that VOCs can be detected and measured non-invasively from breath samples of preterm and full-term infants. In addition, the study showed that full-term infants express different concentrations of VOCs than preterm infants.

Measuring the VOCs from infants’ exhaled breath samples non-invasively is novel. VOCs have been traditionally measured in blood, urine and stool. However, there are limitations related to sampling availability, possibility of cross contamination, and the burden of sample’s processing [10–15]. Non-invasive breath sample has several advantages, such as being always available, could be collected at any time, multiple times a day, potential for real-time results, and avoid multiple blood sampling or obtaining urine or stool. Collecting fecal samples from newborn infants has some difficulties due to the lack of regular samples available when infants, especially preterm infants and especially if they are on parenteral nutrition, do not necessarily defecate every day. Therefore, multiple attempts were made to measure VOCs from exhaled breath. Subjects in these attempts were intubated and supported with invasive mechanical ventilation. A single-center study examined tracheal aspirates from intubated preterm infants using a sensor of electronic nose system [16]. However, one of the limitations of this device is the lack of sensitivity; and when simultaneously measuring multiple compounds, detection limits could be challenging [17]. In another study, VOCs were measured in intubated infants using the High-Performance Liquid Chromatography (HPLC) by nano-HPLC coupled to high-resolution Mass Spectroscopy (MS) [18]. Although in adults, multiple studies measured VOCs successfully in breath non-invasively, we could only find a single recent study that measured them in preterm infants non-invasively using the Gas Chromatography–Mass Spectroscopy (GC–MS) device [19]. The current study measured VOCs non-invasively in preterm and full-term infants. This study used the SIFT-MS for analysis. SIFT-MS is relatively newer compared to the GC–MS. SIFT-MS allows real-time absolute quantification of several trace gases simultaneously, even when an abundance of atmospheric gas is present [20] and that might be the reason that, in this study, the device equally detected VOCs in samples of respiratory circuit and of ambient air of the incubator. Of note, GC–MS is relatively expensive and requires highly trained operators [17].

In the current study, we intended to measure VOCs in preterm infants <30 weeks of GA, because this specific preterm population is liable to complications of prematurity and tends to adopt alternative physiological processes and metabolic pathways that might differ significantly from full-term infants. We also included a control group of full-term infants who were ≥37 weeks of GA to validate the detection and measuring processes. There was a significant difference between both groups for certain compounds, specifically, the 2-propanol, acetaldehyde, acetone, acetonitrile, benzene, ethanol, isoprene, pentane, 3-methylhexane, 2-nonene, ethane, triethylamine, and trimethylamine. All these compounds might have potential sources within the body. Isoprene is a marker of cholesterol synthesis, pentane -produced during lipid peroxidation- and ethane increase after tissue injury, ethanol is generated from bacterial metabolism, acetaldehyde is formed after the oxidation of ethanol, methane -produced from gut flora- is an indicator of carbohydrate malabsorption, acetone is produced when the body utilizes fat rather than glucose for energy, 2-propanol is produced from acetone’s reduction, hydrogen sulfide is a by-product of bacterial metabolism in the mouth, benzene and acrylonitrile usually have exogenous sources, and trimethylamine is produced by gut flora [6, 21, 22].

This study has several strengths, it showed the feasibility of detection and measuring VOCs in newborn infants from the exhaled breath using non-invasive method. Furthermore, to our knowledge, this is the first study that showed that full-term infants express different concentrations of VOCs from preterm infants. In addition, the investigator who processed the samples was blinded, therefore there was no operator bias. Nevertheless, given the pilot nature of this study, the sample size was small; that could impose a limitation when comparing groups as the study was not powered for such comparison. However, the study fulfilled its feasibility aim. Although this study was meant to test feasibility of detecting VOCs in non-intubated babies only, we recommend including controls in future studies for comparing infants with vs without breathing tubes. In addition, the following step in future studies would be to ensure reproducibility of this new technique.

In conclusion, it is feasible to measure VOCs in breath samples of non-intubated preterm and full-term infants non-invasively. Full-term infants express different concentrations of VOCs than preterm infants. Further studies are needed to examine the utility of measuring VOCs to identify and monitor neonatal diseases and predict their outcomes.

## Data Availability

The data that support the findings of this study are available from the corresponding author, MAAF, upon reasonable request.
